# The association of family functioning and suicide in children and adolescents: positive behavior recognition and non-suicidal self-injury as sequential mediators

**DOI:** 10.3389/fpubh.2025.1505960

**Published:** 2025-02-17

**Authors:** Xia Li, Jiahe Liu, Yanling Hu, Xi Huang, Yingxin Li, Yuan Li, Zeyao Shi, Ru Yang, Hanmei Peng, Sisi Ma, Xingli Wan, Wei Peng

**Affiliations:** ^1^Department of Neonatology Nursing, West China Second University Hospital, Sichuan University, Chengdu, China; ^2^Key Laboratory of Birth Defects and Related Diseases of Women and Children, Ministry of Education, Sichuan University, Chengdu, China; ^3^School of Mathematics and Statistics, University of Melbourne, Parkville, VIC, Australia; ^4^Department of Health Policy and Management, West China School of Public Health and West China Fourth Hospital, Sichuan University, Chengdu, China; ^5^Department of Nursing, West China Second University Hospital, Sichuan University, Chengdu, China; ^6^Department of Oncology, West China School of Public Health and West China Fourth Hospital, Sichuan University, Chengdu, China; ^7^Research Center for Palliative Care, West China-PUMC C.C. Chen Institute of Health, Sichuan University, Chengdu, China

**Keywords:** adolescent, child, self-injurious behavior, family functioning, suicide

## Abstract

**Background:**

Suicide is a major behavioral issue among children and adolescents, and family functioning potentially influencing its occurrence. Furthermore, positive behavior recognition, as a key component of positive youth development, may act as a mediating factor in the relationship between family functioning and suicide. Non-suicidal self-injury (NSSI) often co-occurs with suicide and may also mediate the impact of family functioning on suicide. Therefore, the aim of this study is to examine the role of family functioning in child and adolescent suicide, with positive behavior recognition and NSSI serving as sequential mediating factors.

**Methods:**

The participants in this study were recruited from the Chengdu Positive Child Development (CPCD) cohort study. The analysis was based on the second round of cross-sectional data from the CPCD cohort. They were required to complete questionnaires that included measures of family functioning, suicide, positive behavior recognition, and NSSI. SPSS 26.0 and Mplus 8.3 were used for descriptive statistical analysis, correlation analysis and mediation effect analysis.

**Results:**

A total of 8,124 participants completed the questionnaires, with an average age of 11.00 ± 2.18 years. The sample comprised 4,195 male and 3,929 female participants. The findings indicate that 17.93% of children and adolescents reported suicide thoughts, 10.06% had formulated suicide plans, and 8.64% had attempted suicide. Poor family functioning shows a significant positive correlation with suicide (*r* = 0.322, *p* < 0.01). The multiple mediation effect of positive behavior recognition and NSSI in the association between family functioning and suicide was statistically significant (*β* = 0.034, 95% CI = 0.027, 0.042).

**Conclusion:**

This study found that poor family functioning is a risk factor for suicide in children and adolescents, with positive behavior recognition and non-suicidal self-injury acting as sequential mediating factors.

## 1 Introduction

Suicide is a major behavioral concern among children and adolescents. According to data from the World Health Organization (WHO), approximately 703,000 people die by suicide globally each year. In 2019, suicide accounted for 1.3% of all deaths, with a global suicide rate of 9 per 100,000 people. Suicide is the fourth leading cause of death among adolescents aged 15–19. The incidence of suicide attempts among preadolescents under the age of 13 is 2.56%. The lifetime suicide mortality rate in the general population was 7.9 per 100,000 children (1). Currently, mental health is a significant issue for children and adolescents in China. The unique familial and societal pressures faced by Chinese youth, including academic stress and youth unemployment, may contribute to their higher mortality rates compared to those in developed countries (2). In China, the incidence of suicide attempts among children and adolescents under the age of 18 is 3.5%, while the incidence of suicide planning is 6.4% (3).

Family functioning plays a crucial role in the physical and psychological development of children and adolescents. According to family systems theory, individuals with better family functioning tend to have fewer emotional and behavioral issues (4, 5). Prior research has identified family functioning as a potential predictor of suicidal behavior (6–9). Moreover, studies have shown that family functioning influences suicidal behavior both directly and indirectly through mediating factors such as hopelessness, depression, acceptance, and a sense of defeat (7–9).

Positive behavior recognition may have a potential association with family functioning. In recent decades, researchers have gradually shifted their focus in youth development from a “deficit-based” approach to a “positive youth development” approach. The deficit-based approach emphasizes correcting behavioral problems in adolescents, often neglecting their developmental potential, whereas positive youth development focuses on nurturing the assets, abilities, and potential of young people (10, 11). Positive behavior recognition refers to the practice of identifying, acknowledging, and reinforcing desirable behaviors in individuals, and is a key construct in positive youth development (12). Positive behavior recognition seeks to reinforce and sustain desirable behaviors by acknowledging and rewarding them (12, 13). Previous research indicates that rewards are generally perceived as more effective than punishments in fostering positive behavior and enhancing performance among Chinese adolescents (14). Family members play a crucial role in providing positive behavior recognition. Thus, we hypothesize that positive behavior recognition may mediate the relationship between family functioning and suicide.

Self-injurious behaviors are actions that cause deliberate harm to oneself, including non-suicidal self-injury (NSSI), suicidal behavior (15). NSSI is closely related to suicidal behavior. Although suicidal thoughts and behaviors frequently co-occur with self-injury, their underlying causes may differ. Previous studies have established a connection between family functioning and NSSI (16). Therefore, this study hypothesizes that NSSI may also mediate the impact of family functioning on suicide.

**Figure 1:**
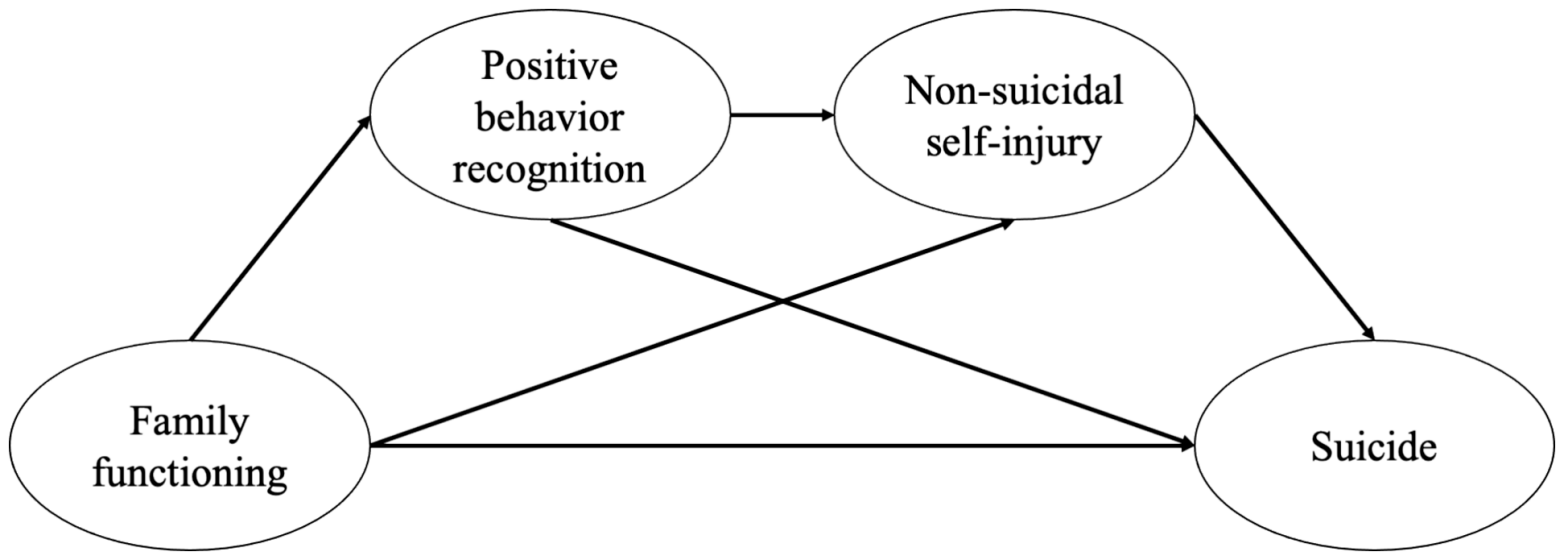
Hypothetical model of the associations among family functioning, suicide, positive behavior recognition, and non-suicidal self-injury.

Thus, this study will examine the role of family functioning in children and adolescents’ suicide, with positive behavior recognition and NSSI serving as mediating factors (Figure 1). In this study, we aimed to conduct a sequential mediation analysis to examine the interrelationships among family functioning, positive behavior recognition, NSSI, and suicide, with the goal of identifying potential pathways for suicide prevention.

The hypotheses of this study are as follows:

*Hypothesis 1:* There is a correlation between family functioning and suicide in children and adolescents.

*Hypothesis 2:* Family functioning affects suicide in children and adolescents, with positive behavior recognition serving as a mediating factor.

*Hypothesis 3:* Family functioning affects suicide in children and adolescents, with non-suicidal self-injury serving as a mediating factor.

*Hypothesis 4:* Family functioning affects suicide in children and adolescents, with positive behavior recognition and non-suicidal self-injury acting as sequential mediators.

## 2 Materials and methods

### 2.1 Participants

The participants in this study were recruited from the Chengdu Positive Child Development (CPCD) cohort study, which has been conducted in Chengdu, the capital of Sichuan province, China, since December 2019. The CPCD study, approved by the Medical Ethics Committee of Sichuan University (K2020025), originally aimed to understand the current state of positive development in children and adolescents, as well as psychosocial and behavioral problems, and to evaluate the effects of Positive Child Development program in promoting positive development and addressing psychological and behavioral issues (17). The first round of surveys for the CPCD study was conducted from December 2019 to January 2020, with the second round taking place from June to July 2020. This cohort study utilized cluster sampling to recruit students from Grades 1 through 9 (ages 6–16) across five primary and secondary schools. These schools were distributed as follows: one in downtown Chengdu, two in the southern suburbs, and two in the northern suburbs. Before participation, written informed consent was given by the students’ legal guardians. This included a project overview, survey procedures, potential benefits and risks and confidentiality agreement. All students provided written informed consent before participating. For this mediation analysis, we utilized data from the second round of the survey.

### 2.2 Data collection

Trained investigators distributed the questionnaires to participants in the classrooms. Before filling out the questionnaires, participants received detailed instructions. They were allowed to ask questions before starting, with investigators providing clarification, but no discussion among participants was permitted during the completion process. The investigators monitored the participants to ensure the questionnaires were completed independently. They also assured participants of the anonymity of the questionnaires to reduce any anxiety or reluctance they may have felt. Participants were allotted 40 to 60 min to complete the questionnaires, and early submission was not allowed to ensure that all responses were fully completed. The questionnaire included several negatively scored items to ensure logical consistency. Any questionnaires with clear logical inconsistencies were excluded from the analysis.

### 2.3 Measures

#### 2.3.1 Family functioning

Family functioning was assessed using the Chinese Family Assessment Instrument (C-FAI), which has demonstrated strong psychometric properties in previous studies (18, 19). The C-FAI consists of 33 items that evaluate five dimensions of family functioning: “communication” (9 items), “mutuality” (12 items), “parental concern” (3 items), “parental control” (3 items), and “conflict and harmony” (6 items). All items were rated on a 5-point Likert scale, with 1 indicating the highest level of agreement and 5 indicating the highest level of disagreement. A higher total score reflects poorer family functioning.

#### 2.3.2 Suicide

Suicide was assessed using three questions: “Have you ever seriously considered trying to commit suicide?” (suicide thought), “Have you made any specific plan to commit suicide?” (suicide plan), and “How many times have you tried to commit suicide?” (suicide attempt) (17). Participants were asked to report how often they experienced each behavior on a 4-point Likert scale: never, 1 time, 2 times, or 3 or more times.

#### 2.3.3 Positive behaviors recognition

Positive behaviors recognition was assessed using a subscale of the Chinese Positive Youth Development Scale (CPYDS), a comprehensive self-report instrument consisting of 80 items across 15 subscales. These subscales include bonding, resilience, social competence, emotional competence, cognitive competence, behavioral competence, moral competence, self-determination, self-efficacy, clear and positive identity, beliefs in the future, prosocial involvement, prosocial norms, spirituality, and positive behavior recognition. The positive behavior recognition subscale includes four items, each rated on a six-point scale: 1 (strongly disagree), 2 (relatively disagree), 3 (slightly disagree), 4 (slightly agree), 5 (moderately agree), and 6 (strongly agree). Higher scores on this scale reflect a greater recognition of positive behaviors. Previous studies demonstrated that the scale has good validity and reliability (10, 20).

#### 2.3.4 Non-suicidal self-injuries

We used the Chinese version of the Deliberate Self-Harm Inventory (DSHI) to assess non-suicidal self-injury (NSSI) behaviors, which was developed and validated by Gratz (21), and later simplified and shortened by Lundh et al. (22). Nine items related to intentional self-harm behaviors without suicidal intent, such as cutting, burning, scratching, biting, and punching, were measured. Participants were asked to report the frequency of these behaviors using a 4-point Likert scale (Never, 1 time, 2 times, and 3 or more times) for each question. A previous study demonstrated that the scale has good test–retest reliability and validity (21, 23). The Chinese version of the DSHI has also been shown to have good reliability and validity and has been applied to children and adolescents in China (24).

### 2.4 Statistical analysis

Statistical analyses were performed using SPSS 26.0 and Mplus 8.3. Missing data were imputed using multiple imputation methods. Descriptive statistics for continuous variables are presented as means and standard deviations (SD), whereas categorical variables are reported as frequencies and proportions. SPSS was used to analyze common method bias and the correlation between variables, while Mplus was utilized for mediation effect analysis. Bootstrapping, a non-parametric resampling procedure, was utilized in this study due to its advantage of not requiring the assumption of normality in the sampling distribution. This method involves repeatedly sampling from the dataset to estimate the effect in each resampled dataset. By performing 5,000 resampling iterations, we generated an empirical approximation of the sampling distribution for the effect of the independent variable on the dependent variable through potential mediators (25, 26). Direct, indirect, and total effects were calculated. Given that the data in this study were self-reported, common method bias may be present. To assess this, Harman’s single-factor test was conducted. A two-sided *p*-value of <0.05 was considered statistically significant.

## 3 Results

### 3.1 Common method bias

Harman’s single-factor test results indicated that 12 factors with eigenvalues greater than 1 were identified, with the first factor accounting for 29.203% of the variance (less than 40%). This suggests that common method bias did not affect the results of this study.

### 3.2 Characteristics and descriptive results

In the second round of data collection, a total of 8,124 questionnaires were completed. Compared to the first round, 701 participants, mostly Grade 9 students, who were unable to participate due to their high school entrance exam preparations, were lost to follow-up. The response rate was 92.06%. The participants had a mean age of 11.00 ± 2.18 years, consisting of 4,195 males and 3,929 females. The findings indicate that 17.93% of children and adolescents reported suicide thoughts, 10.06% had formulated suicide plans, and 8.64% had attempted suicide. Table 1 presents the characteristics and the descriptive results.

**Table 1:** The characteristics and descriptive results (*n* = 8,124).

Characteristics	Mean ± SD	*n* (%)	Median (Min, Max)
Age	11.00 ± 2.18	–	–
Gender			
Male	–	4,195 (51.64)	–
Female	–	3,929 (48.36)	–
Grade			
Primary school (Grade 1–3)	–	1960 (24.13)	–
Primary school (Grade 4–6)	–	3,579 (44.05)	–
Middle school (Grade 7–9)	–	2,585 (31.82)	–
Family functioning	61.51 ± 23.99	–	–
Mutuality	20.10 ± 10.00	–	–
Communication	16.55 ± 8.46	–	–
Conflict and harmony	12.89 ± 5.72	–	–
Parental concern	5.01 ± 2.67	–	–
Parental control	6.95 ± 3.78	–	–
Positive behavior recognition	20.19 ± 3.94	–	–
Suicide			
Suicide thought	–	1,457 (17.93)	–
Suicide plan	–	817 (10.06)	–
Suicide attempt	–	702 (8.64)	–
Non-suicidal self-injury	–	–	0 (0, 27)

SD, standard deviation.

### 3.3 Correlation among variables

Table 2 presents the correlations between variables. Gender and age were included in the analysis to account for their potential influence on the measured variables. The results reveal that gender and age are significantly related to positive behavior recognition, NSSI, suicide, and poor family functioning. Poor family functioning shows a significant positive correlation with both suicide and NSSI (*r* = 0.322, *r* = 0.331, *p* < 0.01, respectively), and a significant negative correlation with positive behavior recognition (*r* = −0.368, *p* < 0.01). Suicide is significantly positively correlated with NSSI (*r* = 0.692, *p* < 0.01) and significantly negatively correlated with positive behavior recognition (*r* = −0.250, *p* < 0.01). Moreover, positive behavior recognition is significantly negatively correlated with NSSI (*r* = −0.250, *p* < 0.01). The relationships between the scores of the five dimensions (mutuality, communication, conflict and harmony, parental concern, parental control) of family functioning assessed by the C-FAI and positive behavior recognition, suicide, and NSSI mirrored the patterns observed between the C-FAI total score and these variables.

**Table 2:** The correlation analysis of family functioning, suicide, the positive behavior recognition, and non-suicidal self-injury.

Variables	Gender	Age	PB	NSSI	SB	FFT	Mut	Com	CH	PCC	PCT
Gender	1										
Age	0.006	1									
PB	0.033**	−0.117**	1								
NSSI	0.056**	0.101**	−0.250**	1							
SB	0.090**	0.111**	−0.250**	0.692**	1						
FFT	−0.025*	0.064**	−0.368**	0.331**	0.322**	1					
Mut	0.01	0.105**	−0.330**	0.308**	0.310**	0.900**	1				
Com	0.034**	0.142**	−0.346**	0.307**	0.322**	0.868**	0.846**	1			
CH	−0.083**	−0.088**	−0.212**	0.183**	0.145**	0.644**	0.353**	0.288**	1		
PCC	−0.069**	−0.006	−0.257**	0.229**	0.200**	0.741**	0.584**	0.536**	0.532**	1	
PCT	−0.089**	−0.052**	−0.184**	0.159**	0.146**	0.524**	0.224**	0.219**	0.615**	0.444**	1

** *p* < 0.01 (two-tailed). * *p* < 0.05 (two-tailed). PB, positive behavior recognition, NSSI, non-suicidal self-injury, SB, suicide, FFT, family functioning, Mut, mutuality, Com, communication, CH, conflict and harmony, PCC, parental concern, PCT, parental control.

### 3.4 Testing of mediation effects

**Figure 2:**
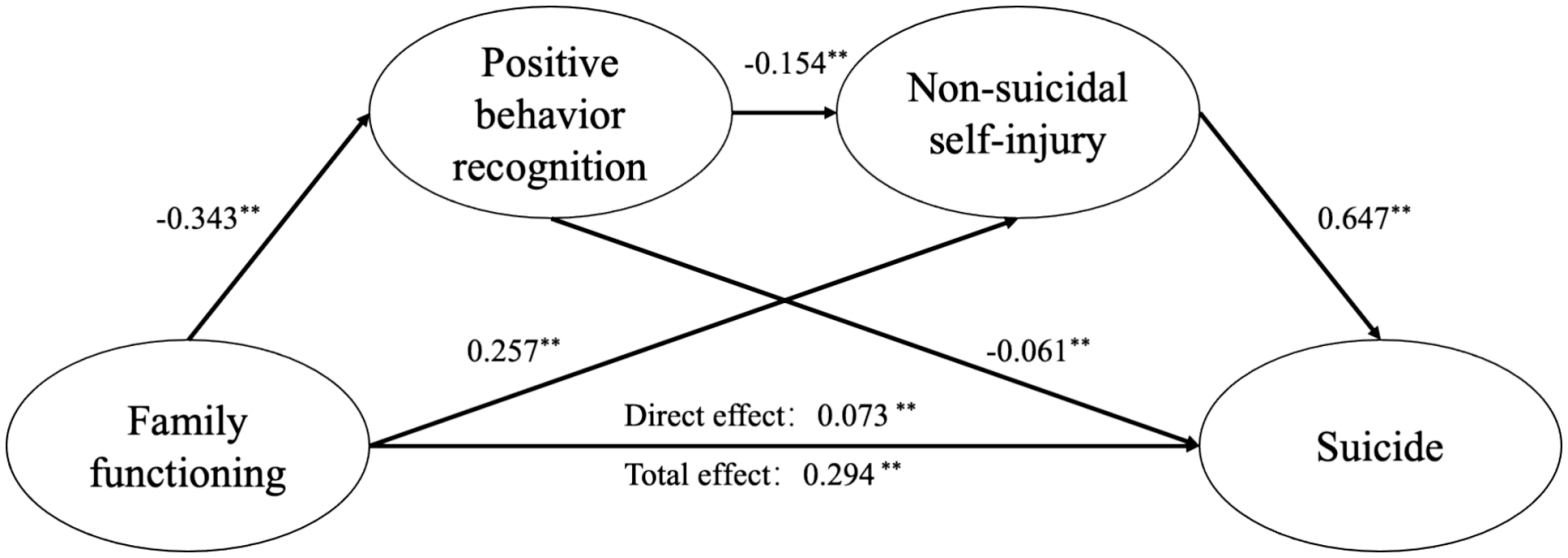
Mediation model of the positive behavior recognition and non-suicidal self-injury on path from family functioning to suicide. ** *p* < 0.001 (two-tailed), adjusting for age, gender.

Mediation analysis was conducted, with age and gender included as control variables. Family functioning was the independent variable, suicide was the dependent variable, and positive behavior recognition and NSSI acted as mediators. The path coefficient results are presented in Figure 2. The mediation analysis revealed that positive behavior recognition significantly mediated the relationship between family functioning and suicide (*β* = 0.021, 95% CI = 0.013, 0.028). Similarly, NSSI was found to mediate this relationship (β = 0.166, 95% CI = 0.149, 0.184). Moreover, the multiple mediation effect of positive behavior recognition and NSSI was significant (β = 0.034, 95% CI = 0.027, 0.042). In total, the indirect effects accounted for 75.17% of the total effect (Table 3).

**Table 3:** Results of mediation analysis with covariates (*n* = 8,124).

Variables	Standardized coefficient	S.E.	95% CI lower	95% CI upper	*p*-value
With covariates (age, gender)					
Direct effects					
FFT → PB	−0.343	0.011	−0.364	−0.322	0.000
FFT → NSSI	0.257	0.012	0.232	0.281	0.000
PB → NSSI	−0.154	0.015	−0.185	−0.124	0.000
PB → SB	−0.061	0.011	−0.082	−0.039	0.000
NSSI→SB	0.647	0.014	0.618	0.675	0.000
FFT → SB	0.073	0.010	0.053	0.092	0.000
Indirect effects					
FFT → PB → SB	0.021	0.004	0.013	0.028	0.000
FFT → NSSI→SB	0.166	0.009	0.149	0.184	0.000
FFT → PB → NSSI→SB	0.034	0.004	0.027	0.042	0.000
Total indirect effects	0.221	0.009	0.203	0.240	0.000
Total effects	0.294	0.012	0.270	0.316	0.000

CI, confidence interval; PB, positive behavior recognition, NSSI, non-suicidal self-injury, SB, suicide, FFT, family functioning, Mut, mutuality, Com, communication, CH, conflict and harmony, PCC, parental concern, PCT, parental control.

## 4 Discussion

The findings of this study reveal a positive association between poor family functioning and suicide in children and adolescents. Specifically, a poor family functioning is linked to an increased likelihood of suicide among this population, thereby supporting Hypothesis 1 (H1). These findings are consistent with those of previous studies (4, 7–9, 27). Based on the correlation analysis, the scores for the five dimensions of family functioning (mutuality, communication, conflict and harmony, parental concern) were all significantly positively associated with suicide. Additionally, Alvarez-Subiela et al. identified specific family factors influencing suicide, including negligence, affectionless control, insecure attachment, and stressful life events as risk factors, while parental care and security serve as protective factors for adolescent suicide (6). This indicates that family interventions could serve as potential strategies for suicide prevention. Enhancing family functioning through improved communication and mutual care among family members may help prevent suicide in children and adolescents. It is also noteworthy that discrepancies exist between children’s and adolescents’ self-reports of family functioning and their parents’ reports (28). Children and adolescents generally report poorer family functioning than their parents do (28). In this study, we used data from the children’s self-reports to avoid any potential misestimation of the relationship between family functioning and suicide that might result from using parental reports.

Family functioning not only directly impacts suicide but also influences it through mediating factors such as hopelessness, depression, acceptance, and so on. This study demonstrates that positive behavior recognition serves as a mediating factor in the relationship between family functioning and suicide, supporting Hypothesis 2 (H2). The goal of recognizing positive behavior is to reward and acknowledge such actions, thereby reinforcing the individual’s positive behaviors (12, 13). There is a negative correlation between poor family functioning and positive behavior recognition. In other words, better family functioning is associated with more effective recognition of positive behaviors. This may be related to the fact that recognition often comes from family members, and families with better functioning are more likely to offer such recognition (12). Additionally, positive behavior recognition has a negative impact on suicide, meaning that it serves as a protective factor against suicide. From a humanistic perspective, positive behavior is indicative of healthy human development. Positive behavior recognition may foster positive youth development by encouraging the maintenance of constructive behaviors, thereby reducing negative behaviors such as suicide and self-injury. The indirect pathway “family functioning → positive behavior recognition → suicide” is established. Specifically, family functioning indirectly affects the risk of suicide through its impact on positive behavior recognition.

This study also confirms that NSSI mediates the relationship between family functioning and suicide, supporting Hypothesis 3 (H3). Previous research has established the influence of family functioning on self-injurious behavior (16, 29–34). Specifically, aspects such as family intimacy and adaptability, the family environment, and family conflict have been found to significantly impact self-injurious behavior (30, 32). The connection between self-injury and suicide is particularly strong. Self-injurious behaviors refer to actions that deliberately inflict harm on oneself (15, 35). NSSI is defined as the intentional destruction or alteration of bodily tissue without suicidal intent, and includes behaviors such as cutting, head banging, burning, hitting oneself, scratching to the point of bleeding, and interfering with wound healing (36, 37). Suicidal behaviors, on the other hand, are self-injurious actions carried out with the intent to end one’s life, such as hanging/strangulation, severe cutting, or jumping from a height (37, 38). Suicidal thoughts refer to the contemplation or planning of actions aimed at ending one’s life (i.e., suicidal ideation or plan) (37). While suicidal behavior and NSSI differ in terms of intent, frequency, and lethality, these behaviors often co-occur (15). Some studies suggest that NSSI or self-harm is a risk factor for suicidal behavior (39–41) and may also serve as a precursor to suicidal behavior (42, 43). The findings of this study confirm that NSSI mediates the pathway between family functioning and suicide, indicating that family functioning indirectly influences suicide through NSSI. Several intervention programs aimed at preventing NSSI have been reported (44–46), suggesting that targeting NSSI prevention may serve as an effective intervention strategy to reduce the risk of suicide.

Research on the link between positive behavior recognition and NSSI is scarce. Nonetheless, the correlation analysis conducted in this study demonstrates a significant negative relationship between positive behavior recognition and NSSI, suggesting that improved positive behavior recognition is associated with a reduced risk of NSSI. Additional analyses using data from this cohort show a significant negative correlation between positive youth development and NSSI, with NSSI potentially serving as a predictor of positive youth development (47). Given that positive behavior recognition is an integral component of positive youth development, the observed correlation between positive behavior recognition and NSSI can be interpreted within this context. Positive behavior recognition and NSSI function as sequential mediators in the pathway from family functioning to suicide. The mediation effect is confirmed, supporting Hypothesis 4 (H4). Theoretically, targeting any point within the mediation pathways could impact suicide outcomes. The demonstration of sequential mediation highlights potential intervention targets for addressing suicide.

The findings of this study highlight the importance of focusing on children and adolescents experiencing poor family functioning, as this group is at a higher risk for suicide. Additionally, suicide prevention efforts should emphasize comprehensive interventions that strengthen family functioning and promote positive behavior recognition. Given China’s family-centered cultural context, family-based interventions should be a core component of suicide prevention efforts, as families play a pivotal protective role in safeguarding the mental health and well-being of children and adolescents. Moreover, the results indicate that the mediating effect of NSSI represents the largest proportion of the total effect, suggesting that NSSI may serve as a key mediator that requires focused attention. Furthermore, this study utilized self-reported data from participants, which may not fully capture or distinguish between NSSI and suicide, given the potential overlap between these behaviors. As a result, there may be an overestimation of the mediating effect of NSSI in the association between family functioning and suicide. Therefore, caution is warranted in the interpretation of these findings.

An additional point to note is that the second round of data used in this study (collected in June–July 2020) coincided with the COVID-19 pandemic. Given the significant stressors related to the pandemic, such as lockdown measures, heightened family stress, and alterations in social interactions, it is likely that family functioning and mental health were affected. Consequently, the study’s findings may differ in non-pandemic situations, suggesting that these results should be interpreted with caution.

## 5 Limitations

This study utilized data from round 2 of the CPCD cohort study for analysis but did not conduct a longitudinal analysis of the results from rounds 1 and 2. Consequently, no conclusions can be drawn about the longitudinal relationship between family functioning and suicide. The CPCD cohort is still ongoing, and it is anticipated that future longitudinal analyses will offer evidence regarding the relationship between family functioning and suicide. Additionally, when examining the impact of family functioning on suicide, there may be other potential confounding variables (e.g., mental health status, family history of mental disorders) beyond age and gender that could be relevant. This study did not account for these factors, and their influence on the results should be considered.

## 6 Conclusion

Among children and adolescents, family functioning has a significant impact on suicide, both directly and indirectly. Positive behavior recognition and NSSI serve as mediators, with both individual and sequential mediation effects being confirmed. Considering the substantial influence of family functioning on suicide, we emphasize the importance of family-based interventions in suicide prevention for children and adolescents. The mechanisms underlying the influence of family functioning on suicide are complex, and, in addition to the known mediators, other potential mediators or moderators may exist and require further investigation.

## Data Availability

The raw data supporting the conclusions of this article will be made available by the authors, without undue reservation.
